# Periscope of Dermatology

**Published:** 1901-02-23

**Authors:** 


### PERISCOPE OF DERMATOLOGY.

**Treatment by X Rays and Light.**—SCHOLEFIELD[1] records the case of a boy, aged 18, who had suffered from severe lupus of the nose for two years, during which time he had been treated by ointments and scarification. After four months' treatment with the X rays all signs of the disease had disappeared. A coil giving an 18-inch spark was used, and the rest of the face was protected with a rubber mask: each sitting lasted a quarter of an hour, and they were repeated every other day for two months, then stopped for a month, and then repeated on alternate days for another month. Considerable desquamation, resembling that resulting from sunburn, was caused.

Hahn and Albers-Schönberg[2] report 12 cases of lupus treated with X rays: of these 7 were cured and the rest greatly benefited. The number of applications of the rays varied from 5 to 58. Rarely in the first stage the lupus nodules actually increased in size, and the surrounding skin became hyperæmic. This, however, did not influence their final disappearance.

Pusey[3] refers to 40 cases of removal of superfluous hairs by means of the X rays. A very weak but definite current will remove the hairs from an entire area. There are no unpleasant symptoms beyond a slight erythema or pigmentation, which, however, only lasts a short time. The treatment is not so tedious as electrolysis, and moreover enables us to remove down as well as thick hairs. It is also useful in producing the preliminary epilation which is required in the treatment of mycotic diseases of the hair.

Schiff[4] recommends the surrounding parts to be covered with sheets of pasteboard covered with lead foil $\frac{1}{15}$ inch in thickness, so as to protect them from the X rays. He has had good results in lupus, in the removal of superfluous hairs, furunculosis, acne, eczema, and elephantiasis. The Roentgen tube is 15 cm. from the skin, and sittings are held daily lasting at first five and later 10 or 25 minutes each. In hypertrichiasis 17 to 25 sittings are required, while in sycosis and favus from 7 to 13 sittings only are necessary. The resulting erythema is cured by a 15 per cent. boracic lanolin.

Morris[5] has employed Finsen's concentrated light method for lupus and for rodent ulcer; he also had good results in lupus erythematosus, although the reaction was very intense even after one application, but it quieted down after a week, and a wide atrophic scar resulted. In contrasting the effects of X rays with those of light, he thinks that the former is quicker, but sometimes gives rise to a troublesome dermatitis, and the healing is more slow than with electric light.

Drigalski[6] experimented with a 15-candle power incandescent lamp, and found these rays quite useless therapeutically. They cause excessive perspiration and symptoms of exhaustion. The bad symptoms are caused by the red rays, which should therefore be excluded from any light bath.

Stenbeck[7] reports the cure of a rodent ulcer by X rays. The rays were applied for 10 minutes daily. After 35 sittings the ulcer was more healthy. The duration of the sittings was increased to 15 minutes, and a month later epidermis had grown over the ulcer.

Kessler[8] employs an incandescent lamp surrounded by a parabolic reflector, which is applied until perspiration occurs. After this the part should be protected with cotton wool. In cases of hæmatoma the pain is quickly relieved, rapid absorption of blood takes place, and no blue mark is left.

Hall Edwards[9] has treated three cases of lupus by means of X rays, and obtained good results, although the ulcer in one case took 12 months to heal. In another case the lupus patch sloughed, and the resulting ulcer was very troublesome. The third case, which was lupus erythematosus, was quite satisfactory.

**Eczema.**—A discussion[10] on eczema took place at the International Congress in Paris, and special attention was given to the question of the parasitic origin of the disease. No definite conclusions were arrived at, but the majority of dermatologists do not regard eczema as a parasitic disease due to a specific organism, while several observers consider that the early eczema vesicle is amicrobic; on the other hand, most are agreed that in the later stages of eczema staphylococci and streptococci play an important part in the evolution of the lesions. Finally there is no consensus of opinion among dermatologists as to the dermatoses which ought to be included under the term eczema.

Whitfield[11] examined the small dry discs of eczema which affect the cheeks of young children. A careful bacteriological examination demonstrated the invariable presence of a peculiar coccus, usually a diplococcus. Inoculative experiments proved negative. The organism was not found in the healthy skin of the face, nor in the vesicular eczema of that region.

Torok[12] discusses the parasitic origin of eczema, and has found staphylococci, streptococci and bacillus subtilis, but considers that these micro-organisms only modify the disease, and that there is no serious argument in favour of the parasitic origin of eczema.

Spiegler[13] recommends the following treatment in those cases of chronic local eczema which extend slowly and recur again and again after healing. The eczematous patches are rubbed with a swab, dipped in a 50 per cent. caustic soda solution. After washing with water a 50 per cent. solution of lapis divinus or copper sulphate is painted on. A bandage is applied over cotton wool, and healing takes places in about two weeks. One application is usually sufficient; but another will be needed if the soda solution has not been properly rubbed in.

Hutchinson[14] recommends the use of a mixture of liquor carbonis detergens and liq. plumbi subacetatis in nearly every form of eczema. A teaspoonful of each to a pint of warm water is commonly ordered, and this is not only employed as a wash, but kept constantly applied on lint. An undiluted mixture may be applied to chronic patches and pruriginous affections. It stings at the moment of application, but does not cause smarting afterwards.

Martyn[15] insists on the necessity of producing a healthy action of the skin and avoiding loss of heat in the treatment of gouty eczema. He prefers cotton cellular clothing to wool. The general health requires attention, but anti-gout remedies are of no value. Locally an ointment containing 10 minims of liq. carbonis detergens in an ounce of lanolin answers well. When there is much irritation baths sometimes do good, a lime-sulphated water being specially good. After the bath the patient must be carefully dried and powdered all over, and great care taken to avoid catching a chill.

**Therapeutics.**—*Epicarin*,[16] which is a product of creosotine acid and naphthol, is recommended in the treatment of scabies and prurigo. Five or six applications are the most which are required. The following ointment is recommended:—℞ Epicarin 115 grs., cret. alb. 30 grs., vaseline 1 oz., lanolin ½ oz., adipis porci 1½ oz. M. f. ung. Epicarin is apt to make the skin dry, brittle, and cracked, and so it is recommended to apply the remedy at night, and in the morning to rub in some unguentum diachyli.

*Petrosulfol*,[17] which is a sulphur compound similar to ichthyol in its chemical and physical action, is useful in eczema, especially in that form known as eczema keratoides. Excellent results also are obtained in pustular eczemas, and sycosis, and in furunculosis. It may be used in the form of an ointment or paste, the following being good formulæ:—(1) ℞ Petrosulfol 4 parts, ung. casein 20 to 40 parts. (2) ℞ Petrosulfol, lanolin, vaseline flavi, āā 20 parts; zinc oxide, amyli āā 10 parts. M. fiat past. It has the power of inhibiting the growth of staphylococci and streptococci; and, moreover, is of value in cases of chronic hyperæmia, chilblains, sweaty feet and hands. No irritating symptoms were noticed.

*Petrolan*[18] is a mixture of mineral oil with soap, and is a brownish-black ointment substance which can be almost completely rubbed into the skin. It has proved of use in eczema. It has strong antiseptic and drying actions, and does not cause irritation or dermatitis. It is spread on the skin and covered with a bandage. Before applying a new bandage the patient should have a warm bath.

Unna[19] points out that linen is unsuitable in skin diseases. If an ointment is spread upon linen, it becomes absorbed by the fabric and does not act on the affected part. It is much better to rub the ointment into the skin and then cover the part with cotton wool, or, better still, with flannel. The avoidance of linen is of still greater importance in the treatment of psoriasis by chrysorobin, where patients must wear pure wool next to the skin.

[1] Brit. Med. Jour., May 5, 1900, p. 1083. [2] Brit. Med. Jour. Ep., April 14, 1900, p. 289. [3] Brit. Med. Jour. Ep., July 28, 1900. No. 74. [4] Brit. Med. Jour., May 5, 1900, p. 1082. [5] Clin. Jour., July 25, 1900, p. 209. [6] Brit. Med. Jour. Ep., July 21, 1900, No. 53. [7] Brit. Med. Jour. Ep., July 21, 1900, No. 54. [8] New York Med. Jour., May 19, 1900, p. 791. [9] Brit. Jour. of Derm., March 1900, p. 105. [10] Brit. Jour. of Derm., Sept. 1900, p. 325; Scott. Med. and Surg. Jour., vol. ii., No 3, p. 266; Lancet, Aug. 18, 1900, p. 533. [11] Brit. Jour. of Derm., Oct. 1900, p. . [12] Brit. Med. Jour. Ep., May 5, 1900, p. 351. [13] Ibid. [14] Med. Rec., New York, Sept. 22, 1900, p. 479. [15] Brit. Med. Jour., Oct. 13, 1900, p. 1082. [16] The Therap., Sept. 18, 1900, p. 248. [17] The Therap., June 15, 1900, p. 156. [18] The Therap., July 16, 1900, p. 190. [19] Brit. Med. Jour. Ep., April 7, 1900, No. 266.

